# Characterization of the Mitochondrial Genome of *Cambaroides schrenckii* (Astacidea: Cambaridae) and Its Phylogenetic Implications

**DOI:** 10.3390/genes15121578

**Published:** 2024-12-08

**Authors:** Xuewei Liu, Ben Li, Yan Yang, Jun Zhang, Chunbo Hu, Yuxi Zhang, Jiawang Zhou, Yinlong Liu, Hongyu Qiu, Chunren Wang, Junfeng Gao

**Affiliations:** 1 Key Laboratory of Prevention and Control of Zoonotic Diseases of Daqing, College of Animal Science and Veterinary Medicine, Heilongjiang Bayi Agricultural University, Daqing 163319, China; 19845919553@163.com (X.L.); m15685258867@163.com (Y.Y.); 17399169679@163.com (Y.Z.); 18378465906@163.com (J.Z.); m17805683201@163.com (Y.L.); qiuhongyu95@163.com (H.Q.); chunrenwang@126.com (C.W.); 2 Animal Disease Prevention and Control Center in Huanan County, Jiamusi 154400, China; leebenps@sina.com; 3 Branch of Animal Husbandry and Veterinary of Heilongjiang Academy of Agricultural Sciences, Qiqihar 161005, China; haifeng1982518@163.com; 4 Longsha Zoological and Botanical Gardens, Qiqihar 161006, China; 17382827416@163.com

**Keywords:** *Cambaroides schrenckii*, Astacidea, mitochondrial genome, phylogenetic analysis

## Abstract

Background: *Cambaroides schrenckii* is an endangered freshwater crayfish in China, belonging to the genus Cambaroides, that can act as a complementary host for paragonimus. The objective of this study was to examine the complete mitochondrial genome characteristics and their evolutionary relationships within the Astacidea. Methods: The analysis of gene rearrangements and evolutionary relationships was conducted through the sequencing of the mitochondrial genome of *C. schrenckii*. Results: *C. schrenckii* mitochondrial genome length was 15,572, comprising thirteen PCGs, two rRNAs, 22 tRNAs, and one d-loop region of *C. schrenckii*. The mitochondrial genome of *C. schrenckii* exhibits an A + T content of 69.61% and a G + C content of 30.39%. Among the thirteen PCGs, cytb, nad3, and nad6 have a start codon of ATT, while the other ten PCGs have ATC, ATA, and ATG start codons. All 22 tRNA genes displayed a typical cloverleaf secondary structure. Gene rearrangement analysis showed that seven gene arrangements were identified based on PCGs in the infraorder Astacidea, with type I being the most common. Conclusions: The relationship between the American Cambaridae is closer to Astacidae than the Asian Cambaridae. The present study provides a theoretical basis for further discussions of developmental relationships in the infraorder Astacidea.

## 1. Introduction

Astacidea is classified within the Arthropoda, Malacostraca, and Decapoda orders. The most salient distinction between members of Astacidea and other Decapoda species is the existence of chelae on the first three pairs of thoracic limbs, with the first pair of chelae being larger than the others. Additionally, the thoracic limbs of the last two pairs are simple, except for the fifth step in *Thaumastocheles japonicus*, which may be equipped with tiny pincers [1]. Morphological characteristics were used to identify five principal families within the infraorder Astacidea. These include two families of freshwater crustaceans (Astacoidea and Parastacoidea), Nephropoidea, Enoplometopoidea, and other families with only fossilised species. As of 2022, the families Cambaridae and Astacidae were included in Astacoidea, which contains 677 species; however, the majority are from the family Cambaridae, with about 600 species [2]. Crayfish are not only a source of culinary delight but also a significant contributor to economic well-being.

*C. schrenckii* (Kessler, 1874) (Decapoda: Astacidea: Cambaridae) constitutes an ecologically and economically important species native to Cambaridae and endemic to the Yalu, Heilongjiang, Ussuri rivers, and Khanka Lake in China [3]. *C. schrenckii* prefers to live in water with lower temperatures due to its high tolerance for water quality. Nevertheless, the population of *C. schrenckii* has experienced a notable decline in recent years, largely attributed to the detrimental effects of water pollution. In 2004, *C. schrenckii* was listed as endangered on the China Species Red List (http://protection.especies.cn/chineseredlist/list (accessed on 21 April 2024)) [4]. Furthermore, *C. schrenckii* represents a significant role as an important complementary host of paragonimus, transmitting the disease to infects dogs, cats, and mice in addition to humans. Furthermore, it is present in 22 provinces and cities in China [5,6]. It is believed that approximately one million people are infected globally each year, making it a significant threat to public health security [7,8].

The identification and classification of substances represents the foundation of all research endeavours. The accurate classification and identification of substances can provide fundamental data for the genetic evolution of corresponding diseases, quantitative genetics, and scientific prevention and control. The mitochondrial genome is a relatively simple, short DNA molecule that evolves rapidly [9,10]. It contains a wealth of genetic information in short sequence fragments, rendering it an optimal molecular marker [11,12]. In the past few years, developments in high-throughput sequencing have provided a more convenient method for studying the complete mitochondrial genome. This method is commonly utilised in the areas of phylogeny, population genetics, and systematics in freshwater crustaceans [13,14]. The phylogenetic and phylogeographic relationships of Austropotamobius (European freshwater crayfish) have been revealed based on mitochondrial *cox*1 sequences [15,16,17]. Using geological proof, phylogeographic studies, and sequences from five genetic regions to investigate the species limits and phylogeographic framework of the freshwater crayfish subgenus Aviticambarus (Cambaridae: *Cambarus*) revealed patterns of cave colonisation. Furthermore, alongside the three currently recognised taxa, two well-supported cryptic species were identified. Four taxa displayed low levels of current and historical genetic diversity, possibly indicating that local extinction events in crayfish are associated with geological and watershed changes [18]. A study of the molecular phylogeny and species classification analysis of all known lobster species in the Pánuco River basin based on the mitochondrial genes 16S rDNA, 12S rDNA, and *cox*1 was conducted [19,20].

Despite the importance of crayfish in the field of zoology, the mitochondrial genome of the Cambaridae family has been the focus of less research than other crustaceans. To date, only the mitochondrial genome sequences of 18 species within this family have been entered into the GenBank database. This lack of research has significantly impeded the development of molecular systematics of crayfish. Therefore, the mitochondrial genome of *C. schrenckii* was first determined. Comparisons and analyses of genome sequences of other crayfish species belonging to different families of the infraorder Astacidea have been published. Gene rearrangements were conducted, and phylogenetic trees were constructed for the infraorder Astacidea to provide a reference for the taxonomic status of *C. schrenckii*. The findings of this study not only enhance the mitochondrial genome data, but also offer novel insights into the classification and phylogeny of Astacidea. Furthermore, they provide an efficient approach to utilising resources for the phylogenetic study of crayfish.

## 2. Materials and Methods

### 2.1. Collection Sample, Extraction of Genomic DNA, and PCR Amplifications

*C. schrenckii* was gathered from Shuangyashan City (46°19′ N, 132°11′ E), Heilongjiang Province. The study protocol was reviewed and approved by the Heilongjiang Bayi Agricultural University Animal Ethics Committee on 11 March 2024 (Approval No. DWKJXY2024027). The sample was carefully cleaned in physiological saline solution, morphologically recognised in accordance with the available description, fixed with 75% ethanol, kept at −20℃, and observed within 48 h. As per the guidelines provided by the manufacturer, the DNA was extracted from a sample muscle tissue using TIANamp genomic DNA kit (TIANGEN Biotech, Beijing, China). Subsequently, the concentration and purity of the proposed DNA were determined using a NanoDrop spectrophotometer 2000 (NanoDrop Technologies, Wilmington, DC, USA). Then, *C. schrenckii* genomic DNA was enhanced by means of PCR reaction. The total volume was 25 μL, and the DNA sample included 1 μL and 2× Tks Gflex PCR buffer (Mg^2+^, dNTP plus) (Takara, Dalian, China) at 12.5 μL. Each primer (10 pmol/μL) was synthesised by Qingke Biotech (Harbin, China) at 0.5 μL, ddH_2_O 10 μL, and Tks Gflex DNA Polymerase (1.25 U/μL) (Takara, Dalian, China) at 0.5 μL. The amplification reaction system was as follows: 95 °C for 1 min; 98 °C for 10 s; 50–64 °C for 30 s; 68 °C for 1 min for 35 cycles; and 72 °C for 7 min. PCR products were measured in 1.0% agarose gel and were ethidium bromide-stained. Molecular identification was conducted by increasing the *cox*1 sequence. The primers were F (5′- GAG CCT CCG TTG ACC T -3′) and R (5′- TGT GAT AAA CAC CGA CCA -3′).

### 2.2. Mitochondrial Genome Sequencing and Assembly, Gene Annotation, and Sequence Analysis

*C. schrenckii* mitochondrial genome high-throughput sequencing was performed using the Illumina NovaSeq platform, and 3.2 GB of NGS data were collected with a read length of 150 bp. Quality control was carried out on the data after sequencing, and linker sequences were filtered. Data pruning mainly included the following points: (1) AdapterRemoval (Version2) was adopted for joint pollution removal at the 3′ end [21]. (2) Mass filtering was carried out by sliding window method with the window size set to 5 bp. Slide the window from the 3′ end to the 5′ and calculate the average Q value of the base in the window; if the Q < 20, delete the base in the window. (3) For length filtering, if the length of any reads in the double end is S 50 bp, the double-end reads are removed. (4) For fuzzy base N filtering, if the number of N bases in the double end is greater than or equal to s, the double-end reads are removed. After quality control, *C. dauricus* (GenBank OL542521) from the same genus served as the reference genome for sequence comparison, and the double-ended data were input into SPAdes v3.15.4 and GetOrganelle v1.7.7.0 for assembly [22,23]. Once the boundaries of the protein-coding genes were determined, they were used in an online analysis through ORF finder (https://www.ncbi.nlm.nih.gov/orffinder/ (accessed on 21 April 2024) ). Then, the 13 PCGs started, the termination codons in the mitochondrial genome of *C. schrenckii* were identified using ORF Finder, and the position and secondary structure of tRNA were predicted using tRNAscan (http://lowelab.ucsc.edu/tRNAscan-SE/ (accessed on 21 April 2024)) [24]. The structure and function were annotated using the MITOS online service (http://mitos2.bioinf.uni-leipzig.de/index.py (accessed on 21 April 2024)) [25]. The annotation results were manually corrected in Geneious R11. CGView [26] server software was used to generate and manually modify the mitochondrial map of *C. schrenckii*. We obtained relative synonymous codon usage (RSCU) for PCGs using MEGA X under the trematode mitochondrial genetic code pattern [27].

### 2.3. Mitochondrial Genome Characteristics and Comparative Analyses

Besides the mitochondrial genome sequence of *C. schrenckii*, the mitochondrial genomes of 42 species (22 Astacoidea species, 2 Enoplometopoidea species, 4 Nephropoidea species, and 14 Parastacoidea species) were obtained from GenBank (Table S1). The nucleotide compositions of ten crustaceans belonging to seven genera of the same superfamily were analysed using Editseq software. Composition skews were calculated according to the formulae AT-skew = [A − T]/[A + T] and GC-skew = [G − C]/[G + C] [28].

By comparing the ratio ω nonsensical substitution rate (dN) and the synonymous substitution rate (dS) of the *cox*1 gene in ten crustaceans of Astacoidea, we evaluated what kind of natural selection the gene experienced: dN/dS (ω) < 1 represented a negative/purified choice; dN/dS (ω) = 1 represented neutral evolution; and dN/dS (ω) > 1 represented a positive/diversified choice. The selection pressure of mitochondrial genes was measured by the average value of the ratio ω between the dN and dS between pairs of sequences, and the ω value between pairs of sequences was computed in DnaSP version 6.12.1 [29]. The selection pressure (dN/dS) of ten species of Astacoidea superfamily crustaceans was calculated using DnaSP version 5.0. The 13 PCGs in the mitochondrial genome of 5 species of Astacoidea crustaceans were serially connected and analysed using DnaSP version 5.0. They were compared to 6 *Cambaroides* species of the 13 PCGs nucleotide and amino acid sequences.

### 2.4. Phylogenetic Analysis

A phylogenetic tree was reconstructed for Astacidea, including 43 species. *Chiromantes haematocheir* (GenBank accession No. NC_042142.1) served as an outgroup. In phylogenetic analysis, we used MAFFT version 7.471 to align the amino acids of 13 PCGs, as indicated in PhyloSuite [30]. A phylogenetic relationship was reconstructed using maximum likelihood (ML) and Bayesian inference (BI) methods. We used phyML version 3.0 to rebuild the ML tree by performing 100 bootstrap duplicates [31]. Bayesian inference phylogenies were inferred using MrBayes version 3.2.6 [32]. The phylograms were drawn using iTOL version 5.0 (https://itol.embl.de (accessed on 21 April 2024)).

## 3. Results

### 3.1. C. schrenckii Mitochondrial Genome Size and Organisation

**Figure 1:**
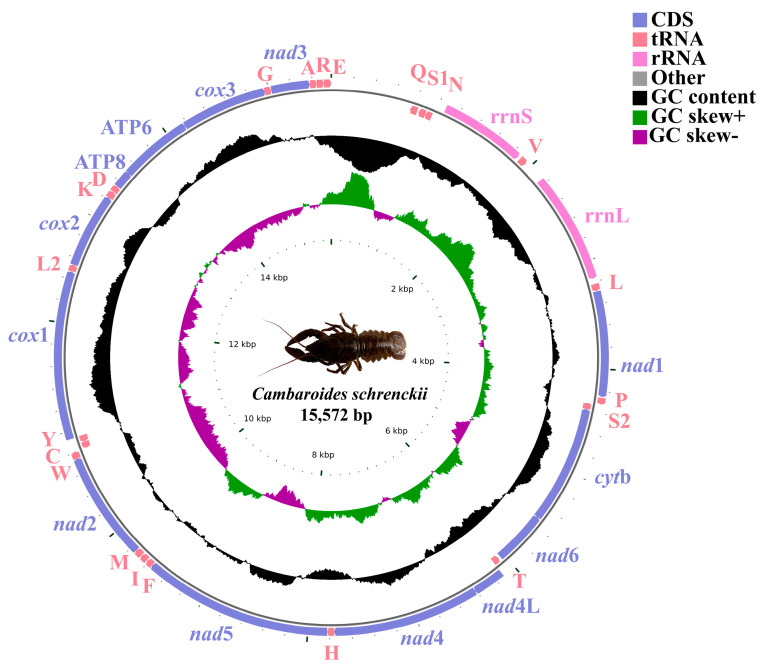
*C*. *schrenckii* mitochondrial genome arrangement. PCGs are shown as blue arrows, rRNA genes as purple arrows, tRNA genes as red arrows, and control region as grey arrows. Ticks in the inner cycle indicate the sequence length. The black ring indicates the GC content (outward and inward peaks showing above or below average GC content, respectively). The purple-green ring indicates the GC skew [(G − C)/(G + C)], purple (between 0 and 1), green (between −1 and 0)].

**Figure 2:**
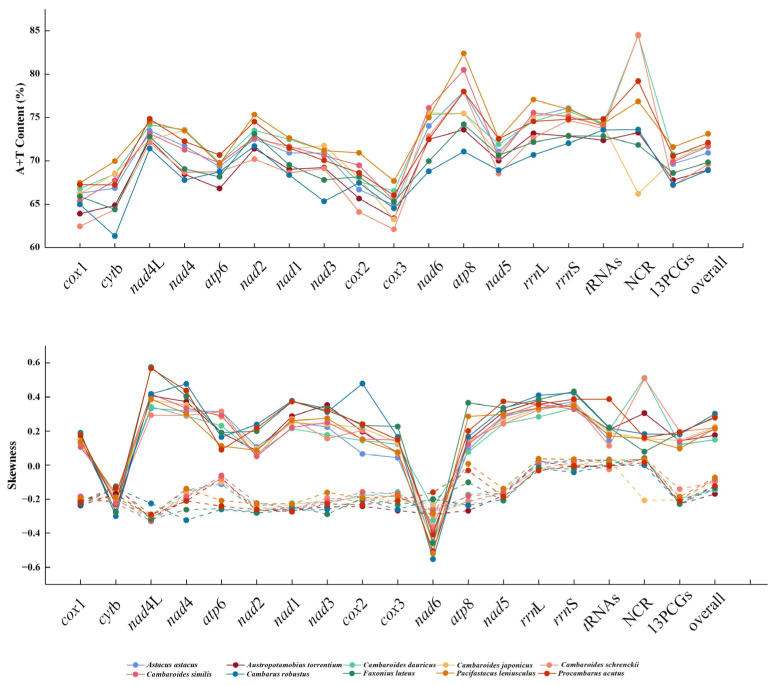
A + T content and nucleotide skew of genes, individual elements, and the complete mitochondrial genome of ten Astacoidea.

The *cox*1 sequencing of *C. schrenckii* from Heilongjiang province showed 100% nucleotide identity when compared to *C. schrenckii* from Russia (GenBank accession No. KX268737). Therefore, the species obtained in this study was identified as *C. schrenckii*. The *C. schrenckii* (GenBank accession No. PP497825) mitochondrial genome was 15,572 bp in length, which is below average compared to other species in the infraorder Astacidea (Figure 1). The *C. schrenckii* complete mitochondrial genome had typical circular molecules featuring 13 PCGs, 2 rRNAs (12S rRNA and 16S rRNA), 22 tRNAs, and a d-loop (764 bp) located between *trn*E and *trn*Q. Nine genes (*nad*2, *cyt*b, *trn*Q, *trn*S1, *trn*N, *trn*S2, *trn*T, *trn*C, and *trn*Y) were encoded on the negative strand, with twenty-eight genes encoded on the positive strand (Table 1). The *C. schrenckii* genome nucleotide composition was biassed towards A + T, with A + T bases comprising 67.18% of PCGs, 72.66% of *rrn*L, 74.68% of *rrn*S, and 73.75% of tRNAs, with the overall genome A + T content being 69.61% (Figure 2). The A + T content of the d-loop was 84.53%, and the C + G content was 15.47%.

### 3.2. Protein-Coding Gene

**Figure 3:**
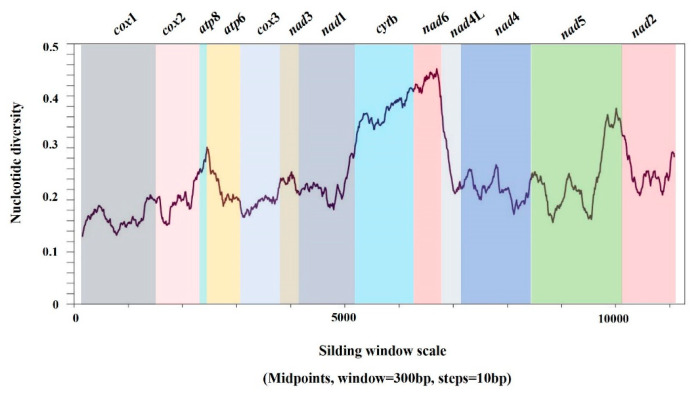
Sliding window analysis of the complete mitochondrial genome sequences of five Astacoidea. A sliding window of 300 bp (in 10 bp overlapping steps) was used to estimate nucleotide diversity Pi (π) across the alignments. Nucleotide diversity was plotted against the mid-point positions of each window. Each gene boundary is identified.

The overall length of the 13 concatenated PCGs for every *C. schrenckii* mitochondrial genome was 11,174 bp, accounting for 71.76% of the entire mitochondrial genome sequence. The length of the 13 PCGs ranged from 159 bp (*atp*8) to 1730 bp (*nad*5). In this standard arrangement of 13 PCGs, all genes related to proteins, except for *cyt*b and *nad*6, were in the positive direction. To identify ten well-conserved and variable mitochondrial genes from the Astacoidea species, the concatenated nucleotide thirteen PCG sequences were analysed using a sliding window (Figure 3). By calculating the quantity of variable positions per unit length of gene, the analysis revealed that *cox*1 was the least variable gene, whereas *cyt*b, *nad*5, and *nad*6 exhibited significant sequence divergence (Figure 3). The evolution rate of the *cox*1 gene for ten species of Astacoidea crustaceans is shown in Table 2; the ω values of the *cox*1 gene for ten species of the Astacoidea superfamily crustaceans are all less than one. *C. schrenckii* is under the greatest selection pressure (ω = 0.02) and does not easily have non-synonymous mutations. In terms of the nucleotide variations in sequence between the mitochondrial genes of the six species of *Cambaroides*, the range was from 0.3 to 22.0%. The total amino acid sequence differences for the 13 PCGs ranged from 0 to 10.3%. The nucleotide sequence variation in the 13 PCGs ranged from 0 to 11.4%, and the amino acid sequence differences ranged from 0 to 15.2% (Table 3).

**Figure 4:**
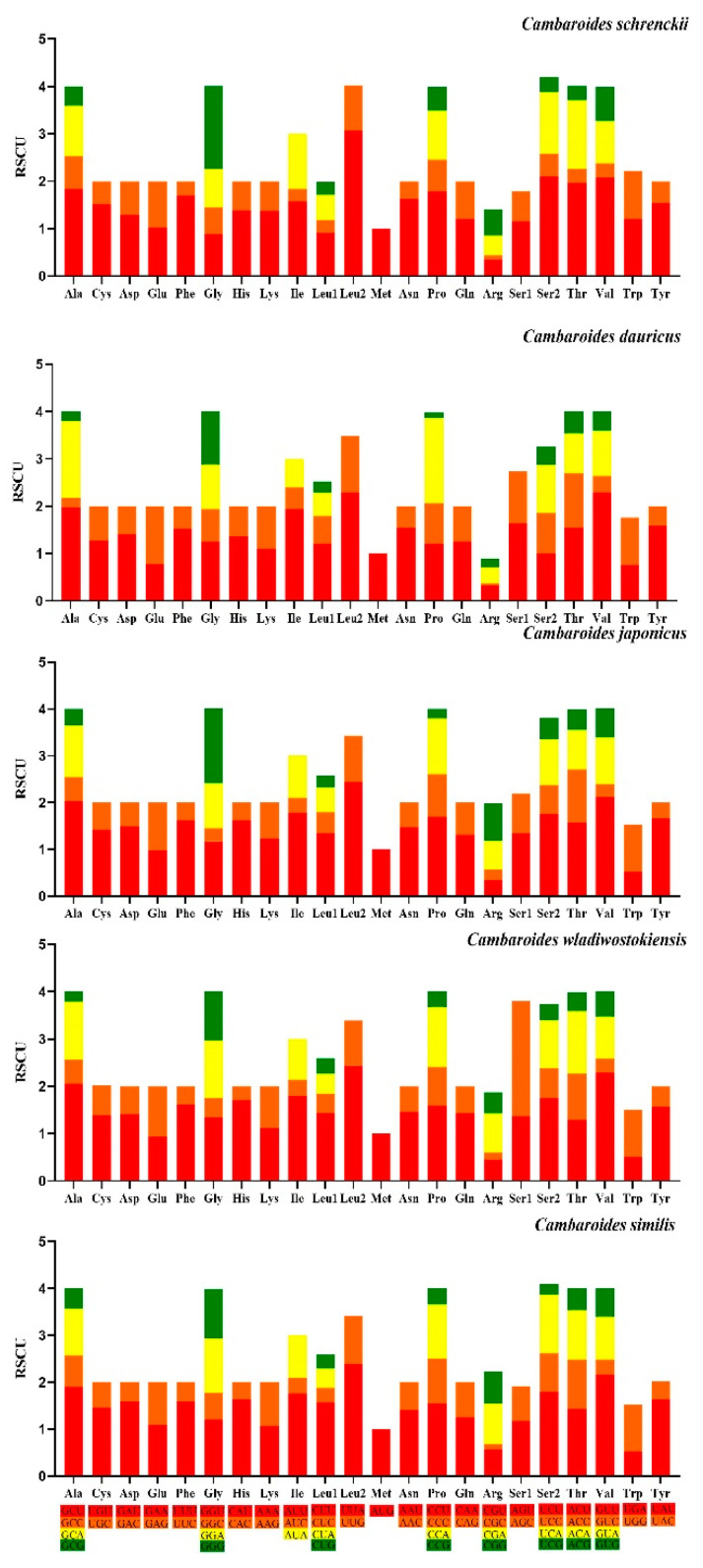
Relative synonymous codon usage (RSCU) of 13 protein coding genes of *C*. *schrenckii*, *C*. *dauricus*, *C*. *japonicus*, *Cambaroides wladiwostokiensis,* and *C*. *similis*. The termination codon is not given.

The RSCU values for the 13 PCGs are displayed in Figure 4. The mitochondrial genome consists of 3724 codons except for start and termination codons. Among the 13 PCGs, Ser2 was the most commonly used amino acid followed by Leu2, Ala, and Gly, while Met was used the least. The total AT-skew and GC-skew of the 13 PCGs were −0.1411 and 0.1966, respectively. Among the 13 PCGs, ATG (8/13) was the most common start codon and TAA (9/13) was the main termination codon (Table 1). The most frequently used codons in the 13 PCGs were ACU (*trn*S2) followed by UUG (*trn*L2) and GGG (*trn*G). The codons CUC (*trn*R), CUG (*trn*L1), and AUC (*trn*I) were used less frequently.

### 3.3. Transfer RNA and Ribosomal RNA

The complete mitochondrial genome of *C. schrenckii* includes 22 tRNAs, the size of which is 61–70 bp. The overall length of the tRNA was 1417 bp, considering 9.11% of the mitochondrial genome. The A + T content in the tRNA region (73.75%) was 2.81 times the G + C content (26.25%). The AT-skew value was −0.026, and the GC-skew value was 0.113. All tRNA sequences can be arranged in the standard cloverleaf structure (Figure S1). Besides the composition of normal base pairs, the clover secondary structure’s stem also includes many non-Watson–Crick base pairs. The most frequent wobble mismatches are G-U wobble base pairs (31 in total). Additionally, 12S rRNA and 16S rRNA were appraised in the heavy chain. rrnS was positioned between *trn*N and *trn*V, and *rrn*L was positioned between *trn*V and *trn*L1. These two genes were separated by *trn*V. The 16S rRNA measured 1017 bp, while the 12S rRNA was 790 bp in length. The A + T contents of *rrn*S and *rrn*L were 74.68% and 72.66%, respectively, and the AT-Skew was 0.0136 and −0.0176, respectively.

### 3.4. Gene Rearrangements

**Figure 5:**
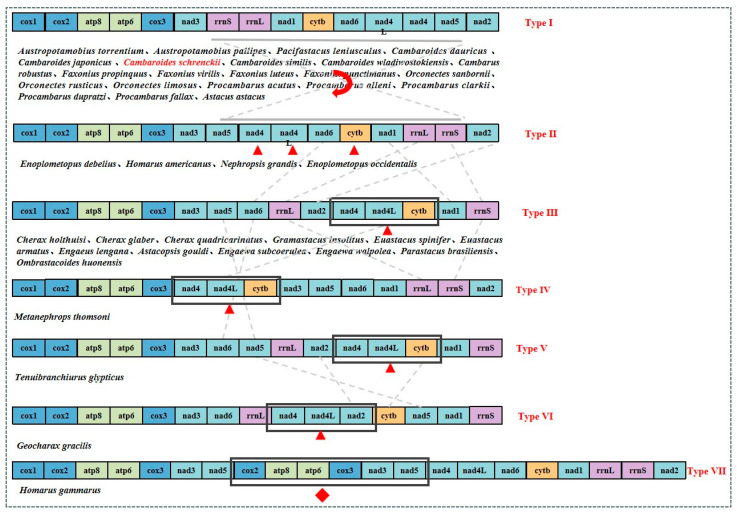
Comparison of the mitochondrial genome organisation of infraorder Astacidea. The circular mitochondrial genome was linearized at the 5′ end of *cox*1 gene for illustration purpose. Non-coding regions were not shown. Red triangular represent gene or gene fragments positional change. Red rhombus represent the duplicated gene fragments. Positions of the inverted blocks are shown with thick grey solid lines. Inversion is specified by the rotating arrow with red lines. The transposition route is indicated by a dashed line.

Gene rearrangements occur frequently in the crayfish mitochondrial genome and were used to examine phylogenetic relationships. *C. schrenckii* rearrangements were investigated by comparing the complete mitochondrial genome of forty-two species from four superfamilies in the infraorder Astacidea. The comparison was based on thirteen PCGs and two rRNAs, resulting in the identification of seven types (Figure 5). The sequenced mitochondrial genomes of 22 crayfish species including *C. schrenckii* in the present study were type I, and the 13 PCGs and 2 rRNA genes had the same arrangement. For the type II gene arrangement, *Enoplometopus debelius*, *Homarus americanus*, *Nephropsis grandis*, and *Enoplometopus occidentalis* had the same arrangement, while the type I inversion occurred in a sizable section enclosed by *rrn*S and *trn*F. For the type III gene arrangement, 12 species had the same gene arrangement. In contrast to type II, large-scale gene rearrangements occurred from *nad*5 onwards in type III, as evidenced by the displacement of *nad*6, *rrn*L, and *nad*2 downstream of *nad*5, *nad*4, *nad*4L, and *cyt*b, which formed a cluster of genes displaced downstream of *nad*2. Additionally, *nad*1 and *rrn*S were displaced downstream of *cyt*b. For type IV, the genotype was compared to type III, with *nad*4, *nad*4L, and *cyt*b forming a cluster of genes that shifted upstream of *nad*3, while *nad*1 shifted upstream of *rrn*L and *nad*2 shifted downstream of *rrn*S. Compared to type III, type V demonstrated a genotype that alternated between *nad*5 and *nad*6. Compared to type V, type VI exhibited a gene cluster comprising *nad*4, *nad*4L, and *nad*2, which was shifted downstream of *rrn*L. In contrast, *cyt*b was relocated between *trn*W and *trn*F, while *nad*5 was relocated between *trn*F and *trn*M. These findings contrasted with the gene arrangement observed in type V. Types VII, VII, and II had the same gene arrangement, except for a large number of repetitive regions.

### 3.5. Phylogenetic Analyses

**Figure 6:**
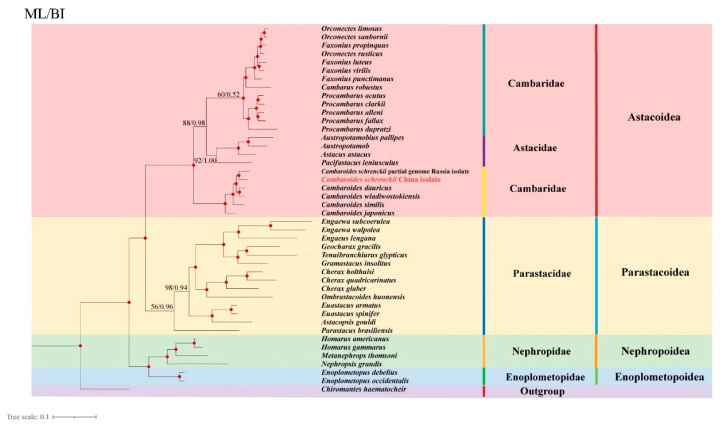
Phylogenetic relationships of *C*. *schrenckii* with other 42 representative Astacidea based on concatenated amino acid sequences of 13 protein coding genes analysed by maximum likelihood (ML) and Bayesian inference (BI) using *C*. *haematocheir* as the outgroup. Red dots on nodes indicate BPP = 100/1.00.

Based on the 13 PCGs, we constructed a phylogenetic tree by selecting species of the four superfamilies Astacoidea, Enoplometopoidea, Nephropoidea, and Parastacoidea as the ingroup, and *C. haematocheir* as the outgroup. A phylogenetic tree was built using the 13 PCGs (Figure 6). The phylogenetic analysis revealed that the ML and BI trees have a largely consistent topology. The four superfamilies (Enoplometopoidea, Nephropoidea, Astacoidea, and Parastacoidea) were divided into four large clades. Notably, Enoplometopoidea and Nephropoidea cluster together and survive in marine environments, while Astacoidea and Parastacoidea cluster together and survive in freshwater environments. In Astacoidea, Cambaridae and Astacidae cluster together, but *Cambaroides* form a separate branch. The relationship between the American Cambaridae is closer to Astacidae than the Asian Cambaridae. *Cambaroides* are classified as a single group, while *C. schrenckii* and *C. dauricus* form separate branches. *C. dauricus* is closer to *C. wladiwostokiensis* than *C. schrenckii*.

## 4. Discussion

The length of the mitochondrial genome of *C. schrenckii* was 15,572 bp. Compared to other Astacidea species, the mitochondrial genome of *C. schrenckii* was smaller than average (Table S1). The d-loop’s overall length determined primary variations between the species, which contained transcription and replication initiation and control signals for the mitochondrial genome [33,34]. The length of different short tandem repeat sequences within the NCR exhibits variability [35]. The A + T content of the d-loop was 84.53% and the C + G content was 15.47%, indicating a strong preference and antisense adenosine phenomenon. These results are consistent with the gene arrangement pattern of the mitochondrial genome of crustaceans in general [36]. This study comprehensively examined the structural features, nucleotide composition, codon preference, and PCG content of the *C. schrenckii* mitochondrial genome. The results indicate a significant antisense cytosine bias in *C. schrenckii*. The total length of all 13 PCGs in *C. schrenckii* was 11,174 bp, constituting 71.76% of the overall mitochondrial genome length. The length of the PCGs ranged from 159 bp (*atp*8) to 1730 bp (*nad*5). In this typical arrangement of the 13 PCGs, all other PCGs were oriented in a positive direction, except for *cyt*b and *nad*6. Comparing PCGs across multiple species can provide insights into their molecular evolutionary patterns and mechanisms [37].

The rate of evolution and nucleotide diversity of genes are often used to gauge the degree of conservation in biological studies [38,39]. A sliding window analysis of conserved and variable mitochondrial gene sequences of thirteen PCGs from five species of Astacoidea revealed significant differences in the thirteen PCGs across ten species of Astacoidea. The variable sites curve indicated that *cox*1 exhibited the lowest variation, whereas *cyt*b, *nad*5, and *nad*6 displayed higher sequence differences. The substitution rate of mitochondrial genes was calculated for all ten species of Astacoidea. The *cox*1 gene was observed to exhibit a ω value less than 1 in all instances, indicating a high degree of interspecific conservation. *C. schrenckii* was found to be most subject to selection pressure (ω = 0.02), indicating that it is unlikely to undergo non-synonymous mutations. The results indicate that the *cox*1 gene may be a useful genetic marker for studies in population genetics and species identification within Astacoidea.

The RSCU values indicated that, with the exclusion of the start and termination codons, the *C. schrenckii* mitochondrial genome has 13 PCGs comprising 3724 codons. The most commonly used amino acids in these PCGs were Ser2, Leu2, Ala, and Gly, while Met was used less frequently. Priority codons were typically associated with crucial functional gene regions, as they were found to favour those with silent sites, which were believed to be associated with maximising translation efficiency [40]. The A + T content was found to be the highest in the third codon position. The PCG findings indicated that the mitochondrial genome of *C. schrenckii* tends to use codons encoding T-rich amino acids. Nevertheless, the codon usage bias for the mitochondrial genome of freshwater crayfish remains uncertain [41].

The *C. schrenckii* mitochondrial genome of the species comprises 22 tRNAs and is folded into the standard cloverleaf secondary structure, with base substitutions or mismatches occurring during the folding process. The base variations in the stem area are fewer than those in the loop area, indicating high conservation. A common belief is that G-U/U-G non-Watson–Crick pairs are less regular and consistent than Watson–Crick base pairs but greater than all other atypical 16 base pairs. Compensatory mutations are essential for functional RNAs as they preserve the RNA structure, thereby contributing to its function. It is traditionally believed that compensatory mutations are achieved through a two-step replacement of G-U (or A-C) base pairs, which are intermediates [42,43].

The gene arrangement indicated that forty-three Astacidea species belonged to four superfamilies, which were divided into seven types in the present study. This study found type I to be the most common arrangement observed in most species of crayfish including *C. schrenckii*, which is consistent with the majority of pan-crustacean arrangements when inversion was disregarded [44]. Some aquatic organisms have been shown to have a faster evolutionary rate in their mitochondrial genomes than terrestrial organisms. Some crustacean species in particular may display elevated rates of mitochondrial genome evolution due to their specific ecological habits [45,46,47]. Recent studies have shown that gene cluster duplication is not only evidence of accelerated evolution, but also a key factor in enhancing biological diversity and complexity [48]. In a mitochondrial genome-wide analysis of decapod crustaceans, Hong et al. identified a deletion of the gene *nad*2 in the *Homarus gammarus* specimen belonging to the family Nephropidae [49]. However, to create a whole mitochondrial genome assembly for *H. gammarus*, Han et al. re-sequenced the genome on an Oxford Nanopore Minion flowcell and carried out a long-read-only assembly [50]. The absence of *nad*2 was detected, and a gene cluster duplication was found in *H. gammarus*. Accordingly, we analysed the mitochondrial genome sequences of *H. gammarus* utilising resequenced *H. gammarus* mitochondrial genome sequences, which have been attributed to type VII. Although gene cluster duplication was only present in type VII, complex gene remakes also occurred in other rows, possibly due to the various evolutionary speeds in different species in specific environments. In total, complex types of gene rearrangements can be observed in the infraorder Astacidea.

Based on an independent partitioned phylogenetic analysis of 43 mitochondrial genomes in the infraorder Astacidea, the results showed that the family Cambaridae is not monophyletic. Nevertheless, the Astacidae family monophyly is a challenging issue in contemporary morphological and molecular analyses. In relation to the families Astacidae and Cambaridae, DNA data could not provide a firm location for the genus *Cambaroides* [51]. However, some distinct physical traits that *Cambaroides* and the American Cambaridae representatives Cambarinae and Cambarellinae share suggest a tight phylogenetic link. Moreover, *C. japonicus* postembryonic development is more similar to Astacidae than American Cambaridae [52]. The similarities between *Cambaroides* and Astacidae postembryonic development appear to confirm Ortmann’s (1897) theory that both taxa are closely related [53]. Therefore, the postembryonic development of *C. schrenckii* is more similar to Astacidae than to American Cambaridae. Furthermore, with the ongoing development of maternal care techniques, the same research identifies the Astacidae + Asian Cambaridae as being more closely related to each other than to the more derived North American Cambaridae + Parastacidae [54]. Recent nuclear mitochondrial DNA sequence investigations of freshwater crayfish throughout the world, including all representatives of *Cambaroides* [54,55], support *Cambaroides* distinct position in relation to the North American cambarids and European astacids. Given the body of data, it seems likely that the Asian genus *Cambaroides* will eventually have a distinct family status. Bracken Grissom et al. discovered that the Asian cambarids and astacids form a monophyletic group despite the majority of research supporting the Asian cambarid lineage as being the most foundational within the Astacoidea [56]. They proposed expanding the idea that *Cambaroides* belong to the Astacidae. The data from Frederic et al. strongly support the basal status of the *Cambaroides* lineage, as indicated by the analysis of five Astacide species, two Procambarus, two Orconectes, one Cambarus, and four *Cambaroides*, utilising both nuclear and mitochondrial genes [57]. A taxonomic classification of Northern Hemisphere crayfish at the family level may consider classifying Asian cambarid crayfish as a new family. Northern Hemisphere crayfish should be grouped into a single family, similar to how crayfish from the Southern Hemisphere are treated as Parastacidae members. Our results also indicate that *Cambaroides* constitute a distinct branch, with other species of the family Cambaridae exhibiting a closer relationship to the family Astacidae than to the genus *Cambaroides*.

## 5. Conclusions

The present study sequenced and annotated the mitochondrial genome of *C. schrenckii* for the first time. Gene rearrangement analysis in the infraorder Astacidea identified seven distinct gene arrangements based on mitochondrial PCGs, with type I being the most common. Evolutionary analyses indicated that the relationship between the American Cambaridae is closer to Astacidae than the Asian Cambaridae. The present study provides a theoretical basis for further discussion of developmental relationships in the infraorder Astacidea.

## Figures and Tables

**Table 1:** Mitochondrial genome structure of *C*. *schrenckii*.

Gene	Start	Stop	Size (bp)	Direction	Intergenic Nucleotides	Start Codons	Stop Codons	A + T%
D-loop	1	763	763		-	-	-	84.53
*trn*Q	764	833	70	-	-	-	-	71.43
*trn*S1	848	914	67	-	14	-	-	67.16
*trn*N	915	978	64	-	0	-	-	70.31
*rrn*S	1071	1860	790	+	92	-	-	74.68
*trn*V	1885	1952	68	+	24	-	-	69.12
*rrn*L	2154	3170	1017	+	201	-	-	72.66
*trn*L1	3220	3284	65	+	49	-	-	72.31
*nad*1	3288	4249	962	+	3	ATG	TAG	68.61
*trn*P	4257	4320	64	+	7	-	-	71.88
*trn*S2	4323	4386	64	-	2	-	-	78.12
*cyt*b	4387	5521	1135	-	0	ATT	TAA	64.41
*nad*6	5521	6039	519	-	−1	ATT	TAA	72.83
*trn*T	6060	6122	63	-	20	-	-	79.37
*nad*4L	6125	6418	294	+	2	ATG	TAA	72.11
*nad*4	6418	7761	1344	+	−1	ATG	TAA	68.68
*trn*H	7761	7824	64	+	−1	-	-	84.38
*nad*5	7825	9554	1730	+	0	ATG	T	68.55
*trn*F	9555	9615	61	+	0	-	-	67.21
*trn*I	9621	9684	64	+	5	-	-	73.44
*trn*M	9688	9752	65	+	3	-	-	70.77
*nad*2	9753	10,745	993	+	0	ATG	TAA	70.19
*trn*W	10,745	10,810	66	+	−1	-	-	68.18
*trn*C	10,810	10,872	63	-	−1	-	-	77.78
*trn*Y	10,873	10,934	62	-	0	-	-	70.97
*cox*1	10,932	12,470	1539	+	−3	ATC	TAG	62.44
*trn*L2	12,473	12,536	64	+	2	-	-	67.19
*cox*2	12,537	13,221	685	+	0	ATG	T	64.09
*trn*K	13,222	13,285	64	+	0	-	-	71.88
*trn*D	13,287	13,349	63	+	1	-	-	85.71
*atp*8	13,350	13,508	159	+	0	ATG	TAA	77.99
*atp*6	13,505	14,176	672	+	−4	ATA	TAA	68.75
*cox*3	14,176	14,964	789	+	−1	ATG	TAA	62.10
trnG	14,963	15,024	62	+	−2	-	-	77.42
*nad*3	15,025	15,377	353	+	0	ATT	TAA	69.12
*trn*A	15,379	15,439	61	+	1	-	-	73.77
*trn*R	15,440	15,504	65	+	0	-	-	73.85
*trn*E	15,505	15,572	68	+	0	-	-	80.88

**Table 2:** *Cox*1 gene selection pressure in Astacoidea conformaceae.

	A	B	C	D	E	F	G	H	I
B	0.04								
C	0.06	0.08							
D	0.07	0.06	0.07						
E	0.06	0.05	0.07	0.06					
F	0.05	0.05	0.05	0.06	0.05				
G	0.05	0.07	0.04	0.04	0.02	0.07			
H	0.06	0.04	0.07	0.04	0.03	0.08	0.08		
I	0.06	0.09	0.03	0.06	0.07	0.04	0.07	0.05	
J	0.06	0.04	0.07	0.05	0.06	0.05	0.05	0.05	0.07

Note: A: *Astacus astacus*; B: *Austropotamobius torrentium*; C: *Cambaroides dauricus*; D: *Cambaroides japonicus*; E: *C*. *schrenckii* in present study; F: *Cambaroides similis*; G: *Cambarus robustus*; H: *Faxonius luteus*; I: *Pacifastacus leniusculus*; J: *Procambarus acutus*.

**Table 3:** Identity of nucleotides and predicted amino acids for protein-coding genes in Cambaroides.

Genes	Identity of Nucleotides/Amino Acids (%)				
	CSC/CSR	CSC/CD	CSC/CJ	CSC/CS	CSC/CW
*cox*1	100.0/100.0	95.8/96.1	95.8/96.1	95.2/95.1	95.8/96.1
*cox*2	99.7/100.0	98.7/98.2	99.1/99.1	96.5/94.3	98.8/98.7
*atp*8	100.0/100.0	91.7/88.5	90.4/88.5	91.0/86.5	90.4/88.5
*atp*6	99.7/100.0	92.5/90.0	92.3/90.5	91.3/88.7	92.4/90.5
*cox*3	100.0/100.0	98.2/97.3	98.7/98.1	98.2/97.7	97.8/97.3
*nad*3	100.0/100.0	92.6/86.3	94.0/88.9	93.7/88.9	94.6/89.7
*nad*1	100.0/100.0	95.5/93.0	95.6/93.6	95.0/92.0	96.1/93.6
*cyt*b	100.0/100.0	96.6/95.0	98.1/97.1	95.9/94.2	97.9/96.8
*nad*6	100.0/100.0	93.4/89.5	95.0/91.9	89.9/85.5	93.2/88.4
*nad*4L	100.0/100.0	96.6/94.8	95.2/92.8	95.2/91.8	95.9/93.8
*nad*4	100.0/100.0	94.2/91.5	88.6/84.8	93.6/90.1	94.5/91.9
*nad*5	100.0/100.0	95.5/92.5	96.6/95.1	94.8/91.8	96.1/94.1
*nad*2	100.0/100.0	93.8/90.3	94.5/91.8	91.5/87.0	93.8/91.2
Total	99.7/100.0	95.3/93.0	78.0/89.7	80.6/90.2	81.4/92.1

## Data Availability

The mt genome sequence of *C. schrenckii* generated in this study was deposited in the NCBI GenBank under accession no. No. PP497825. Raw reads of newly sequenced crayfish of *C. schrenckii* were deposited in the public repository BioProject under accession No. PRJNA1163325.

## Supplementary Materials

The following supporting information can be downloaded at https://www.mdpi.com/article/10.3390/genes15121578/s1, Figure S1. Twenty-two tRNA cloverleaf secondary structures of *C*. *schrenckii*. Table S1. List of species used in the phylogenetic analysis.
