# Supplementary material for: Characterization of the Mitochondrial Genome of Cambaroides schrenckii (Astacidea: Cambaridae) and Its Phylogenetic Implications
Source: Genes (Basel). 2024 Dec 8;15(12):1578. doi: 10.3390/genes15121578 (PMC11675430; doi:10.3390/genes15121578)
Supplement: Supplementary file 1 [file genes-15-01578-s001.zip › Table S1.pdf]

Table S1. List of species used in the phylogenetic analysis

Superfamily	Family	Genus	Species	GenBank Number	mt DNA Size (bp)
Astacoidea	Astacidae	*Astacus*	*Astacus astacus*	MT862440	15,179
	Cambaridae	*Austropotamobius*	*Austropotamobius torrentium*	NC_033504	15,330
			Austropotamobius pallipes	NC_026560	15,679
		Pacifastacus	*Pacifastacus leniusculus*	NC_033509	16,331
		Cambaroides	*Cambaroides dauricus*	OL542521	15,580
			Cambaroides schrenckii(in present study)	PP497825	15,572
			Cambaroides schrenckii partial genome(Russia isolate)	KX268737	14,895
			Cambaroides japonicus	KX268736	16,257
			Cambaroides similis	JN991196	16,220
			Cambaroides wladiwostokiensis	OR353741	16,391
		Cambarus	*Cambarus robustus*	NC_033507	15,797
		Faxonius	*Faxonius propinquus*	NC_068681	15,360
			Faxonius virilis	NC_068680	15,859
			Faxonius luteus	KX268739	16,215
			Faxonius punctimanus	KX119150	15,815
			Orconectes sanbornii	NC_029721	15,883
			Orconectes rusticus	NC_029720	15,875
			Orconectes limosus	NC_026561	16,223
		Procambarus	*Procambarus acutus*	NC_033510	15,747
			Procambarus alleni	NC_028447	15,942
			Procambarus clarkii	OL542520	15,937
			Procambarus dupratzi	NC_070242	15,000

			Procambarus fallax	NC_020021	15,253
Enoplometopoidea	Enoplometopidae	*Enoplometopus*	*Enoplometopus debelius*	NC_025592	15,641
			Enoplometopus occidentalis	NC_020027	15,111
Nephropoidea	Nephropidae	*Homarus*	*Homarus gammarus*	NC_020020	14,316
			Homarus americanus	NC_015607	16,432
		Metanephrops	*Metanephrops thomsoni*	NC_027608	19,835
		Nephropsis	*Nephropsis grandis*	NC_073507	15,977
Parastacoidea	Parastacidae	*Astacopsis*	*Astacopsis gouldi*	NC_026215	16,678
		Cherax	*Cherax holthuisi*	NC_026224	15,889
			Cherax glaber	NC_022939	15,806
			Cherax quadricarinatus	NC_022937	15,869
		Engaeus	*Engaeus lengana*	NC_022847	15,934
		Engaewa	*Engaewa subcoerulea*	NC_029407	16,001
			Engaewa walpolea	NC_029395	15,984
		Euastacus	*Euastacus armatus*	NC_026575	15,555
			Euastacus spinifer	NC_026214	15,558
		Geocharax	*Geocharax gracilis*	NC_023810	15,924
		Gramastacus	*Gramastacus insolitus*	NC_030531	15,704
		Ombrastacoides	*Ombrastacoides huonensis*	NC_041154	17,559
		Parastacus	*Parastacus brasiliensis*	MG551495	16,572
		Tenuibranchiurus	*Tenuibranchiurus glypticus*	NC_025647	17,342
Grapsoidea	Sesarmidae	*Chiromantes*	*Chiromantes haematocheir*	NC_042142	15,899
