# Supplementary figures and images for: Characterization of the Mitochondrial Genome of Cambaroides schrenckii (Astacidea: Cambaridae) and Its Phylogenetic Implications
Source: Genes (Basel). 2024 Dec 8;15(12):1578. doi: 10.3390/genes15121578 (PMC11675430; doi:10.3390/genes15121578)

## Slide 1
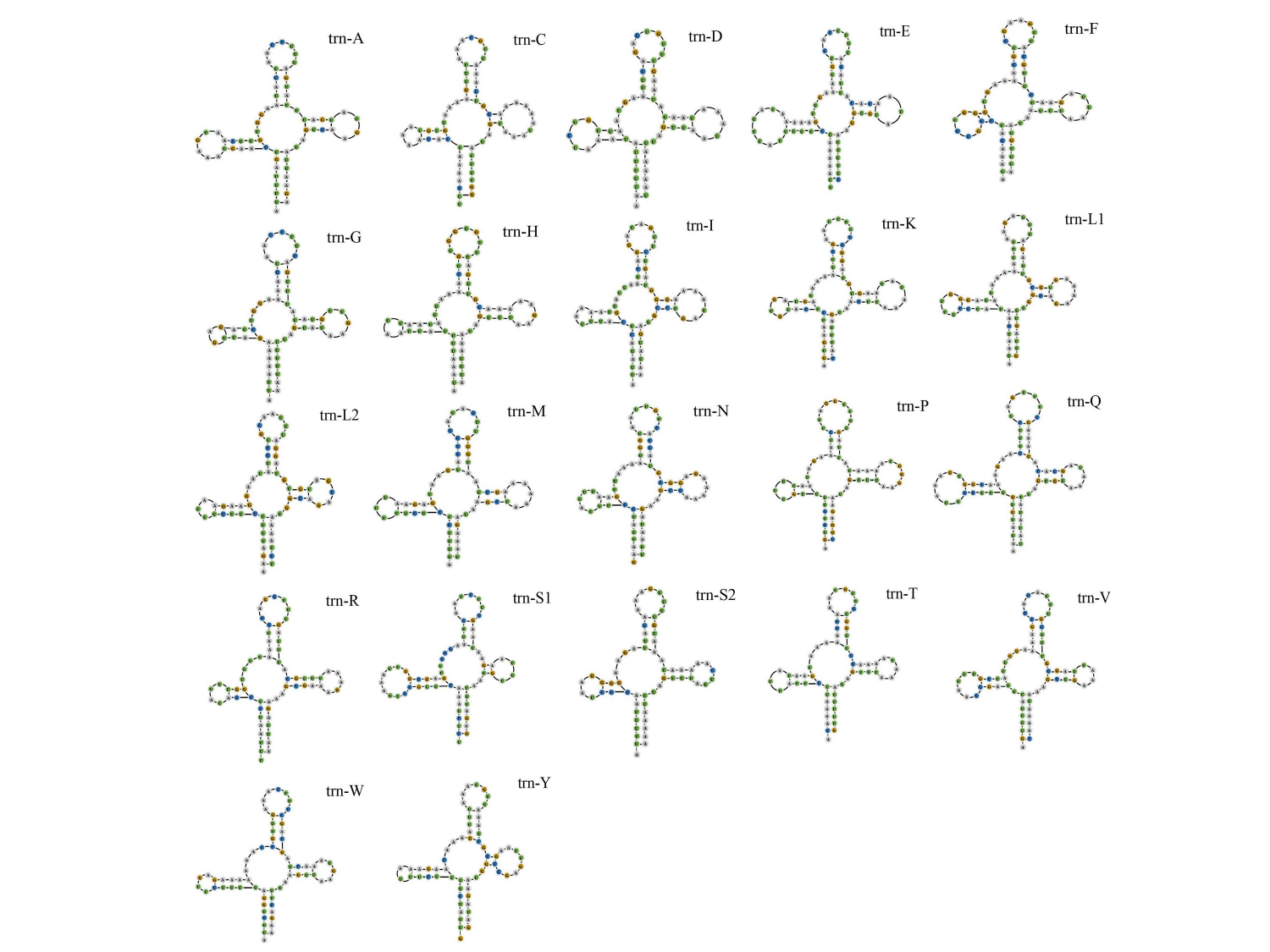

Supplement: Supplementary file 1 [file genes-15-01578-s001.zip › Figure S1.pptx]
